# Something in Our Ears Is Oscillating, but What? A Modeller’s View of Efforts to Model Spontaneous Emissions

**DOI:** 10.1007/s10162-024-00940-7

**Published:** 2024-05-06

**Authors:** Hero P. Wit, Andrew Bell

**Affiliations:** 1 Department of Otorhinolaryngology/Head and Neck Surgery, University of Groningen, University Medical Center Groningen, Groningen, Netherlands; 2 Graduate School of Medical Sciences, Research School of Behavioural and Cognitive Neurosciences, University of Groningen, Groningen, Netherlands; 3 John Curtin School of Medical Research, The Australian National University, Canberra, Australia

**Keywords:** Self-sustaining oscillator, Micro-mechanical element, Cochlear amplifier, Standing wave

## Abstract

When David Kemp discovered “spontaneous ear noise” in 1978, it opened up a whole new perspective on how the cochlea works. The continuous tonal sound emerging from most healthy human ears, now called spontaneous otoacoustic emissions or SOAEs, was an unmistakable sign that our hearing organ must be considered an active detector, not just a passive microphone, just as Thomas Gold had speculated some 30 years earlier. Clearly, something is oscillating as a byproduct of that sensitive inbuilt detector, but what exactly is it? Here, we give a chronological account of efforts to model SOAEs as some form of oscillator, and at intervals, we illustrate key concepts with numerical simulations. We find that after many decades there is still no consensus, and the debate extends to whether the oscillator is local, confined to discrete local sources on the basilar membrane, or global, in which an assembly of micro-mechanical elements and basilar membrane sections, coupled by inner ear fluid, interact over a wide region. It is also undecided whether the cochlear oscillator is best described in terms of the well-known Van der Pol oscillator or the less familiar Duffing or Hopf oscillators. We find that irregularities play a key role in generating the emissions. This paper is not a systematic review of SOAEs and their properties but more a historical survey of the way in which various oscillator configurations have been applied to modelling human ears. The conclusion is that the difference between the local and global approaches is not clear-cut, and they are probably not mutually exclusive concepts. Nevertheless, when one sees how closely human SOAEs can be matched to certain arrangements of oscillators, Gold would no doubt say we are on the right track.

## Early Days

Otoacoustic emissions (OAEs) are weak sounds emitted by the inner ear. They were discovered almost 50 years ago by Kemp [1, 2] as “evoked cochlear mechanical responses” (ECMRs), and in this way, an entire new field of study began.

This paper gives a largely chronological account of diverse attempts to describe spontaneous otoacoustic emissions (SOAEs), based on physical and electronic models. Step by step, we discuss novel contributions, punctuated now and then by numerical simulations whose goal is to illustrate key points. The focus will be largely on human SOAEs, although other laboratory animals also exhibit them, as do birds and lizards. (We refer the reader to reference [3] for information on the generation mechanism of otoacoustic emissions across tetrapod groups).

Before venturing into our modelling journey, it helps to recognise that Kemp’s discoveries were in fact anticipated in 1948 by Gold [4], a physicist who worked on war-time radar and who realised that the ear needed to be active so as to overcome the huge damping effect of the fluids which fill the organ. His suggestion was that the ear operated like a regenerative receiver, a simple radio circuit of the time which could receive and amplify faint signals. Its circuitry was based on a single thermionic valve which, using positive feedback, “regenerated” the received signal so as to improve sensitivity and reduce bandwidth. Most relevantly, if the feedback gain was set too high, the device could be set into continuous oscillation and “squeal”, which strongly reminded Gold of tinnitus. After inducing temporary tinnitus by a very loud sound he placed a microphone in his ears, but his experiment failed to detect a continuous oscillation. It was left to Kemp to find what Gold had been searching for. (See Bell [5] for a more detailed account, and Gold [6] for a fascinating retrospective).

It is of great interest to note that a regenerative receiver, when oscillating, is a kind of oscillator whose topology is very close to that of the Van der Pol oscillator, a device which has inspired many hearing researchers and which we will return to a number of times in the following text. The path is somewhat winding, as research tends to be, but for those with limited time or patience, there are two key take-home messages.

Our first message is that there is still on-going debate about how the cochlea works and how SOAEs are generated, meaning there is currently no “best” model. The debate tends to centre around two classes of models: “local” models in which there are assumed to be discrete oscillating sources on the basilar membrane, and “global” models in which an assembly of tuned micro-mechanical elements and basilar membrane sections, coupled by inner ear fluid, interact. In the global model, the constituent elements are so heavily damped that their impulse response is a damped oscillation, but when they are coupled strongly enough, the configured system produces one or more self-sustaining oscillations.

As will be seen in what follows global models appear to have gained the upper hand over the past decades, but our simulations convince us that there is not much difference between the two approaches for the mammalian cochlea, and indeed that they may not be mutually exclusive. To underline the point, it is clear that if there are multiple oscillating elements in the inner ear, all surrounded by fluid, they must all be coupled to some degree or other, so the distinction can never be absolute.

Another point to keep in mind during our survey is that a pure tone in the ear canal has a physical counterpart on the basilar membrane. This point was recognised by Goldstein in 1967 [7], writing that “each spectral component in a sustained stimulus excites a limited region at its characteristic place along the basilar membrane”. For an SOAE, detected in the ear canal, this means that something is actually vibrating back and forth on the partition at the same frequency as the SOAE. This has been experimentally verified by Nuttall et al. in 2004 [8]. Nuttall and colleagues used a laser vibrometer to detect spontaneous oscillation at 15 kHz on the basilar membrane of a guinea pig, corresponding to an SOAE picked up at that same frequency in its ear canal.

With this preparation, we begin the SOAE story in earnest. Within a few years of Kemp’s startling discovery, it was soon confirmed by others [9–13], and by 1991 Probst et al. [14] could write an extensive review of the different classes of OAEs and their properties. Notably, Probst described a special class of OAEs called spontaneous otoacoustic emissions (SOAEs): narrow-band sounds emitted by the ear in the absence of any acoustic stimulation and which can be detected by a sensitive microphone in the ear canal. The first published report of SOAEs was in 1979, also by Kemp [15]. In September of the same year, Wilson [16] gave a report in the Proceedings of the Physiological Society of recordings using a sensitive microphone of an objective “tinnitus”. A year later, Zurek [17] confirmed that “objective tonal tinnitus” is measurable in the human ear canal, and he was the first to call the phenomenon “oto-acoustic emission” (OAE) [18]. Soon after, Wit et al. [19] reported the presence of narrow peaks in frequency spectra of spontaneous emissions, probably generated by “strong emission generators”. These emissions could be phase-locked by a click, as earlier described by Wilson [13; Fig. 14].  Synchronised spontaneous otoacoustic emissions (SSOAEs), although not recognised as SOAEs at the time, were documented by Wit and Ritsma [9; Fig. 4].

## Enter the Van der Pol Oscillator

**Fig. 1:**
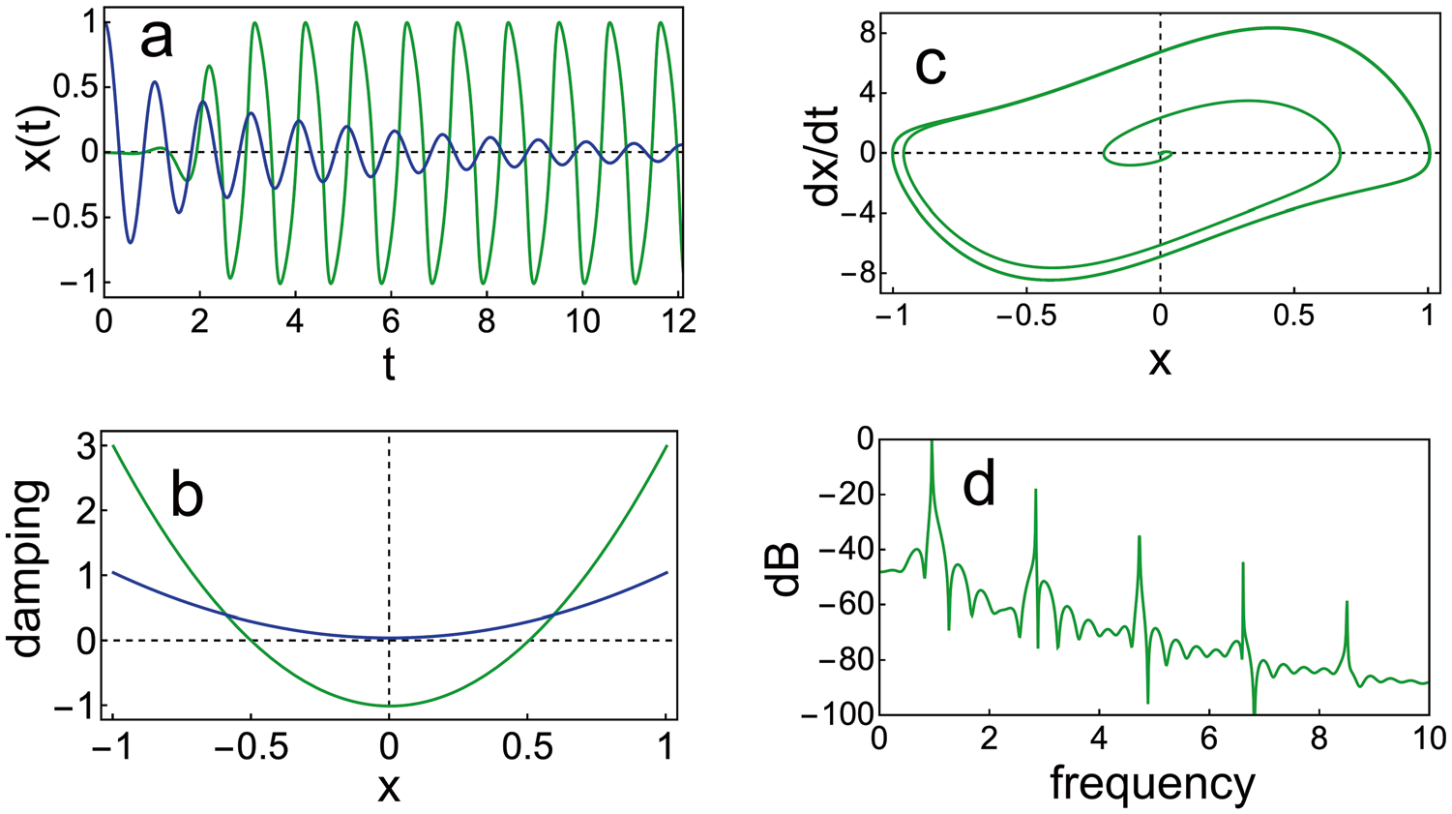
Characteristic behaviour of the Van der Pol oscillator with equation $\ddot{x} +\omega (-\alpha +\beta x^2)\dot{x}+\omega ^2 x = 0$, for $\omega = 2\pi$. **a** Normalised amplitude *x* as a function of time *t*. Green curve, for negative damping for small displacements, creating a permanent oscillation: $\alpha = 1$, $\beta = 4$, $x(0)=0.001$, $\dot{x}(0)=0$; Blue curve, for small positive damping for small displacements, creating an oscillation which slowly dies out: $\alpha = -0.05$, $\beta = 1$, $x(0)=1$, $\dot{x}(0)=0$. **b** Damping profile $-\alpha +\beta x^2$. Green curve: $\alpha = 1$, $\beta = 4$; Blue curve: $\alpha = -0.05$, $\beta = 1$, as in panel a. **c** A plot of velocity versus displacement for the green curve in panel a (the limit cycle). **d** Amplitude spectrum for the green curve in panel a (this curve approaches a pure sine wave for $\alpha \ll 1$)

At the 5th International Symposium on Hearing in 1980, Johannesma [20] proposed the Van der Pol oscillator [21] as a model for the generator of the emissions, essentially replicating Gold’s regenerative receiver. The essential feature of a Van der Pol oscillator is that it has negative damping for small displacements and positive damping for larger ones. Translated to the cochlea, this means it can amplify weak sounds but limit the response to loud ones. This distinctive behaviour is illustrated in Fig. 1.

By assuming that an SOAE is generated by a Van der Pol type oscillator, Long and Tubis [22, 23] gave an explanation of their observation that human SOAEs are suppressed by the consumption of aspirin. Before aspirin consumption, the damping profile of the oscillator has a negative part (as in the green line of Fig. 1b). After consuming the drug, the damping becomes positive for all values of displacement *x* (as the blue line in Fig. 1b). In other words, aspirin consumption changes the oscillator from active (self-sustaining) to passive.

Bialek and Wit [24] investigated the properties of strong human SOAEs by modelling them as a damped mass-on-spring oscillator with active feedback (their Fig. 1). Their equation for the oscillator took the form of a generalised Van der Pol oscillator driven by a random noise force, although here the damping term was slightly modified. Bialek and Wit plotted the probability distribution for the filtered SOAE signal (their Fig. 2), and concluded that the distribution was “essentially what one would obtain from a pure sinusoidal oscillation with a small amount of added noise”. Distinctively, the probability distribution had a minimum of around zero sound pressure.

**Fig. 2:**
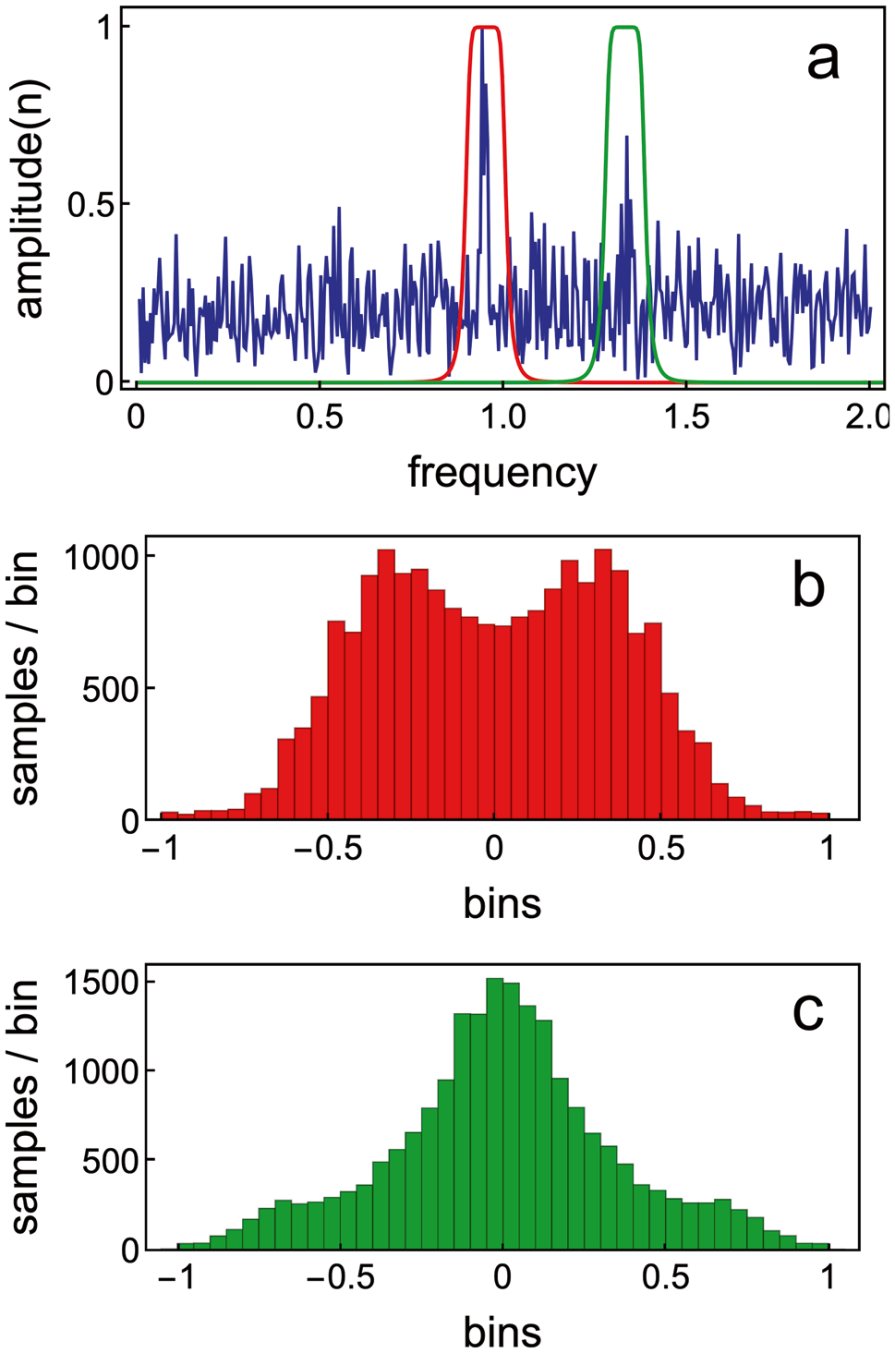
Results of driving a Van der Pol oscillator with narrow-band noise and adding some white noise. **a** Blue: amplitude spectrum of calculated “SOAE”-signal. Red: filter profile around “SOAE”-peak. Green: filter profile around another peak in the noisy signal. **b** Histogram for the numbers in the array $x_{fil}(t), (t = 0, 0.01,0.02,...,20000)$ for the“SOAE”-peak. **c** The same for the noise peak. The message is that an active oscillator will be continually driven away from zero displacement, producing a double-peaked distribution. On the other hand, noise has an amplitude distribution with a maximum at zero displacements

To illustrate this, we again calculate *x*(*t*) for the parameters that produced the green curve in Fig. 1a, driving the oscillator with a $0.5f_0$-wide narrow-band noise (whose centre frequency is the same fundamental frequency $f_0$ shown in Fig. 1d). White noise is added to the resulting sum signal. This mimics an actual SOAE signal, as can be seen in the amplitude spectrum shown in Fig. 2a, where there is a dominant signal peak and a number of noise peaks. We now add a bandpass filter centred around the “SOAE”-peak, as well as around a strong noise peak, and calculate amplitude distributions for each of the filtered signals. If we centre the filter around the SOAE, we get the distribution shown in Fig. 2b, while for the filter around the noise peak, the distribution of Fig. 2c is obtained.

The double-peaked distribution shown in Fig. 2b is a universal property of strong SOAEs, as was demonstrated by Van Dijk et al., who obtained a similar distribution for SOAEs from frog ears [25].

Zwicker and Schloth [26] showed that an SOAE from a human ear can be synchronised, or “frequency locked”, to an external pure tone. This effect was investigated in detail by Long et al. [27] who slowly swept the frequency of a low-level tone across a spontaneous emission. These authors saw that the resulting sound pressure in the ear canal progressed from a region of simple beating to one of relatively constant amplitude (with occasional asymmetric beating), and finally to a region of virtually constant level (without beats) when “frequency locking” or “entrainment” occurred. All of these observations strongly support the notion that SOAEs can be modelled as being generated by a self-sustaining oscillator (requiring a power source) of some kind.

Van Dijk and Wit [28, 29] investigated human SOAEs synchronised to the $f_s=2f_1-f_2$ distortion product of two externally imposed tones. If $f_s$ was sufficiently intense, the SOAE became synchronised (phase-locked) to the distortion product which is generated by the ear’s well-known nonlinearity [7]. For lower levels, the SOAE occasionally slipped out of synchronisation. The authors considered that this behaviour was consistent with a model consisting of a self-sustained oscillator in the presence of weak noise.

By comparing several possible candidates for the role of SOAE generator — including a Van der Pol oscillator — Talmadge et al. [30] also concluded that an SOAE is most probably generated by a noise-perturbed self-sustaining oscillator. The authors again based their conclusion on the spectral and statistical distributions of ear canal SOAEs, and on how they interact with external tones.

Duifhuis et al. [31] constructed a time-domain model of the cochlear partition based on an array of 400 coupled Van der Pol oscillators [32] and showed that small groups of oscillators could be entrained by a relatively strong (30 dB) 1 kHz tone. The natural frequencies of the oscillators in their model decreased exponentially from 20 to 0.1 kHz, and they found that oscillators in the range of 5 to 1 kHz could be entrained.

Van Hengel et al. [33] used the same model, but with a different damping profile for the oscillators (their Fig. 1), to investigate interactions between large numbers of SOAEs. The model was able to explain the observed preferred minimal distances between SOAE frequencies (frequency ratios of about 1.06) in human SOAE spectra [34]. If only one of the 400 sections in the otherwise passive model was made active, the excitation pattern (maximum velocity of a section as a function of its distance from the stapes) showed several sharp minima ([33], Fig. 6). They explained the shape as the result of the active section creating a wave that travels towards the stapes, where it is reflected, because of a mismatch in mechanical coupling between the cochlea and the middle ear. The reflected wave then interferes with the outgoing wave, creating a standing wave pattern. To illustrate this process, we set up an array of Van der Pol oscillators inside a fluid-filled box and numerically calculate what happens when energy is supplied to one of the oscillators to overcome its damping. The non-mathematically inclined reader can skip to the end of the “Intermezzo”.

### Intermezzo 1: An Array of Van der Pol Oscillators in a Fluid-Filled Box

**Fig. 3:**
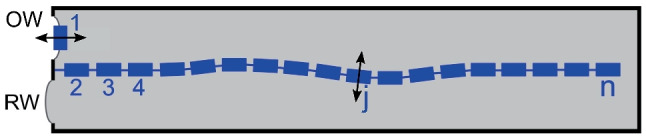
A model of the human cochlea considered as an array of micro-mechanical elements inside a fluid-filled box. The rectangles numbered 2 to n represent the elements, and rectangle 1 is a damped harmonic oscillator representing the structures that transmit vibrations between the cochlea and the ear canal. OW: oval window; RW: round window

Consider a one-dimensional array of $n-1$ micro-mechanical elements immersed in fluid inside a rigid-walled box (Fig. 3). Each element is intended to represent a cross-section of the organ of Corti in the human cochlea.

If elements 2 to *n* in Fig. 3 are Van der Pol oscillators [21], and if each oscillator is driven by fluid pressure only, the equation to be solved (for $j=2$ to *n* for the time course of displacement $x_j(t)$ of the *j*-th oscillator) is the differential equation for a Van der Pol oscillator, driven by an external force:

1 $$\begin{aligned} \ddot{x}_j(t)+\omega _j\gamma (t) \dot{x}_j(t)+\omega _j^2 x_j(t)= \kappa p_j(t) \end{aligned}$$

where damping $\gamma (t)=-\alpha _j +\beta _jx(t)^2$; $\omega _j$ is the natural angular frequency, and $p_j(t)$ the fluid pressure acting on the *j*-th oscillator. Constant $\kappa$ has value 1 and dimension $m^2/kg$ to give both sides of the equation the dimension of an acceleration. Since $\alpha _j$ is dimensionless, $\beta _j$ has the dimension $m^{-2}$. The equation $\ddot{x}_1(t)+d_1\omega _1\dot{x}_1(t)+\omega _1^2x_1(t)=p_1(t)$ is added to the set of Eq. (1). It is the equation for oscillator 1, being a damped harmonic oscillator that represents the middle ear and the ear drum. It is driven by fluid pressure component $p_1(t)$.

The fluid pressure $p_j(t)$ in Eq. (1) is calculated as

2 $$\begin{aligned} \varvec{p}(t)=a\varvec{F}^{-1}\varvec{\ddot{x}}(t), \end{aligned}$$

which applies Elliott et al.’s state space formulation for the human cochlea [35–38]. With $\varvec{p}(t)=\{p_1(t), p_2(t),...,p_n(t)\}^T$ and $\varvec{\ddot{x}}(t)=\{\ddot{x}_1(t), \ddot{x}_2(t),...,\ddot{x}_n(t)\}^T$. See Eqs. (14) and (15) in [37] for $n \times n$ matrix ***F***. The value for pressure parameter *a* $(= 0.2)$ was derived as in Appendix 1 of [39].

The set of $n = 351$ equations was solved with *Mathematica 13* ’s NDSolve routine, for a total time of 50 ms and a time step of 5 $\mu$s.

The parameters for the second-order non-linear damping profile are $\beta _j=20$ and $\alpha _j=-0.1$, for all $j=2$ to *n*, except for oscillator 200. By giving $\alpha _{200}$ the value 0.1, oscillator 200 is a self-sustaining (active) local oscillator; all other oscillators are passive. Initial values were $x_j(0)$ and $\dot{x}_j(0)$ are 0 for all *j*, except $\dot{x}_{200}(0)=0.01$. Other values: damping $d_1 = 0.1$, angular frequency $\omega _1 = 2\pi$ kHz. Natural angular frequencies for oscillators 2 to *n* decreased exponentially from $10\times 2\pi$ to $0.14\times 2\pi$ kHz, giving oscillator 200 a frequency of 0.9 kHz.

**Fig. 4:**
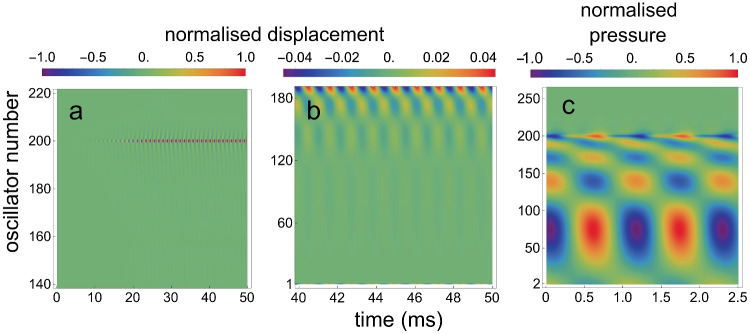
Density plots for the array when it contains one self-sustaining oscillator. **a** For the normalised $x_j(t)$ for $j=$140 to 220, for the situation that oscillator 200 is active. **b** For 40 to 50 ms after $t=0$, for $j=$1 to 187. The maximum amplitude in this panel is 25 times smaller than the amplitude of oscillator 200. **c** For 2.5 ms of the normalised $p_j(t)$ for $j=$2 to 260, starting around $t=45$ ms after $t=0$

Figure 4a and b are density plots for the calculated $x_j(t)$, for different time intervals and for different sets of oscillators. Figure 4c is a density plot for the calculated $p_j(t)$.

By inspection of Fig. 4b and c, it is clear that oscillator 200 has created a standing wave, both in the array of oscillators and in the fluid pressure. The wave is present between oscillator 200 and oscillator 1, but not for $j>200$.

**Fig. 5:**
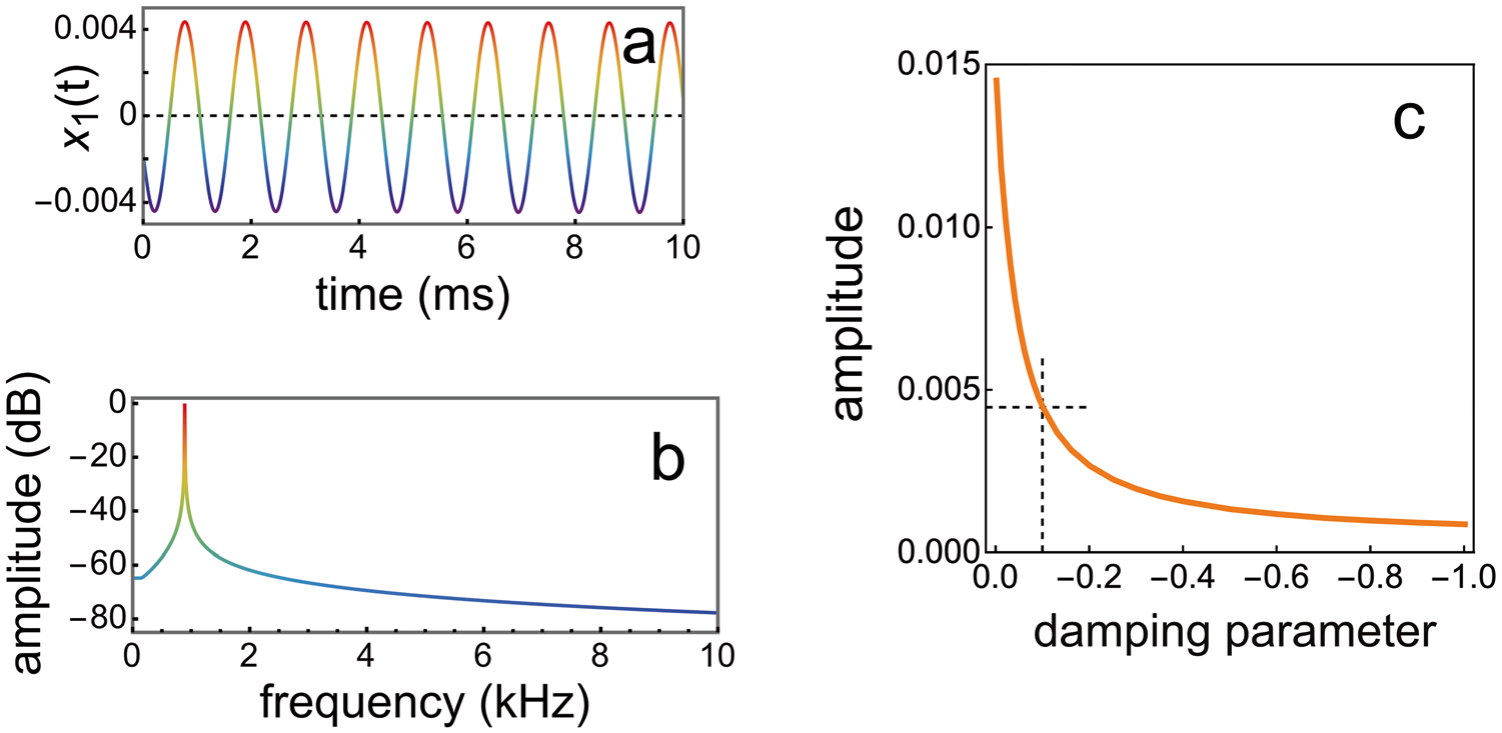
Characteristics of a calculated spontaneous otoacoustic emission. **a** 10 ms of $x_1(t)$. **b** Normalised amplitude spectrum for $x_1(t)$. **c**. Relation between damping parameter $\alpha _j$ and the amplitude of $x_1(t)$. The dashed lines mark the situation shown in panel a

Calculations were repeated for the same set of parameters, but now for a time interval of 300 ms. The last 10 ms of $x_1(t)$, which can be considered to represent (part of) a spontaneous otoacoustic emission, is shown in Fig. 5, together with its amplitude spectrum, calculated for the last 100 ms.

Subsequently, $x_1(t)$ was recalculated for a range of values for damping parameter $\alpha _j$ for all oscillators, except for the active oscillator (where it remained 0.1). The influence of $\alpha _j$ for the passive oscillators on the amplitude of $x_1(t)$ is shown in Fig. 5c: it can be seen that the smaller the damping of the passive oscillators, the larger is this amplitude.

The message from this simulation is that when an array of fluid-coupled oscillators — in which one of the oscillators is active — is connected to the middle ear, this single active oscillator will create a standing wave along the array. The fluid pressure at the high-frequency end of the array will then drive oscillator 1, representing the middle ear and eardrum, and an SOAE will emerge. Another message is that the amplitude of this SOAE depends not only on the amplitude of the active oscillator, it also depends strongly on the (un)damping of the passive oscillators that create the standing wave in the fluid.

***End of Intermezzo 1***

## From the Single Van der Pol to Compound Versions

Van Dijk and Wit [40] found a positive correlation between amplitude and frequency fluctuations in isolated SOAEs in both human ears as well as frog ears. They compared their results with that of a second-order oscillator interacting with a noise source and concluded that an oscillator with linear stiffness (for example a Van der Pol oscillator exposed to white Gaussian noise), cannot account for all experimental results. In particular, the slow frequency fluctuations of measured SOAEs are in conflict with the very rapid frequency fluctuations in the model.

**Fig. 6:**
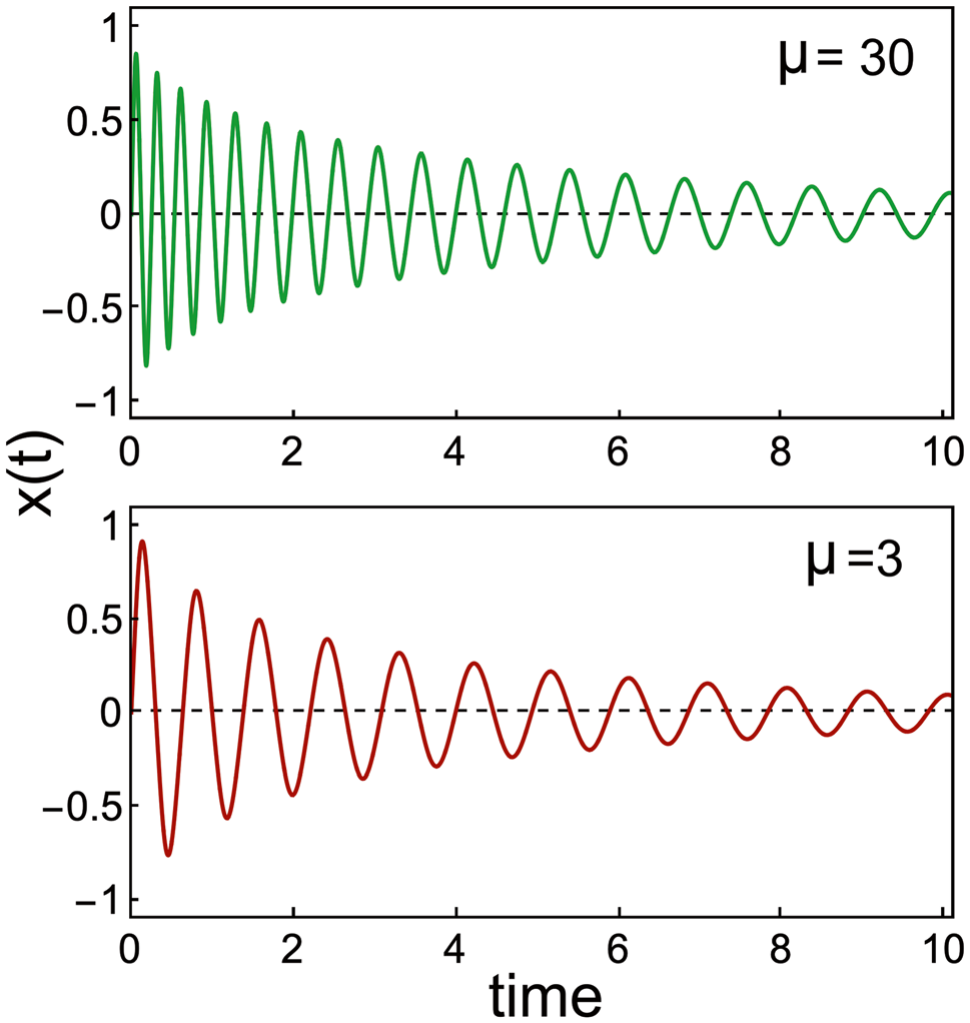
Displacement *x*(*t*) calculated for the Van der Pol — Duffing oscillator with equation $\ddot{x} +\omega (-\alpha +\beta x^2)\dot{x}+\omega ^2 (\lambda +\mu x^2)x = 0$, for $\omega =2\pi , \alpha =-0.05, \beta =1, \lambda =1$, for two values of “spring hardening” parameter $\mu$. Note how for higher values of $\mu ( >0)$ higher frequencies are produced for larger amplitudes

Searching for a more satisfactory formulation, a few years later Van Dijk et al. [41] used a noise-perturbed Duffing oscillator — which is a Van der Pol oscillator with nonlinear stiffness — to explain the correlation between amplitude and frequency fluctuations in SOAEs from seven human subjects. For this type of oscillator, its oscillation frequency depends on its oscillation amplitude, and this is illustrated in Fig. 6. The authors concluded that an even-order nonlinear stiffness plays only a minor role in the generation of SOAEs.

They also saw a clear 1 Hz amplitude modulation in the power spectrum of the emission of one of the subjects. In fact, Bell [42] provided human SOAE data showing this property, and suggested this phenomenon could be caused by heart-beat (his Fig. 5). Amplitude modulation was later specifically investigated by Long and Talmadge [43], who found a strong correlation between the separation of SOAE spectral sidebands and the subject’s pulse rate. They demonstrated that the sidebands must stem from frequency (not amplitude) modulation of the emissions. As a possible explanation, they suggested the modulation could be due to very small changes (10–100 ppm) in the mass of the basilar membrane which accompanies the flow of blood to the cochlea.

As possible candidates for the generation of SOAEs, Talmadge et al. [30] investigated several types of oscillators having nonlinear stiffness, including the Duffing oscillator. They demonstrated that a broad class of noise-perturbed oscillators, with nonlinear stiffness, may account for some, but not all, statistical properties characteristic of SOAEs. Further, they also thought that if data on SOAE interactions with external tones are taken into account, that the evidence for a self-sustaining source of SOAEs is even stronger. Examples of such interactions were extensively investigated by Murphy et al. [44–46], who concluded that their results could be adequately described by an isolated limit-cycle oscillator interacting with an external tone. Murphy et al. [45] also investigated the mutual suppression of SOAEs during the imposition of pulsed external tones. They showed that, instead of a single Van der Pol oscillator, a twin Van der Pol oscillator model, in which two oscillators feed back on each other, can quantitatively describe the dynamical features of this phenomenon. A similar conclusion had been reached earlier by Wit [47], who described the behaviour of two SOAEs which regularly alternated in strength, and showed how such mutual suppression could be modelled in terms of a coupled Van der Pol oscillator model and its electronic analogue.

Sisto and Moleti [48] added an extra term to the equation of the Van der Pol oscillator, given in the legend of Fig. 1 above, to be able to explain “some important aspects of the OAE phenomenology”. One major aspect was the suppression of an SOAE by an external tone and its following recovery within a few tens of milliseconds. The equation for the active nonlinear oscillators in their model was

**Fig. 7:**
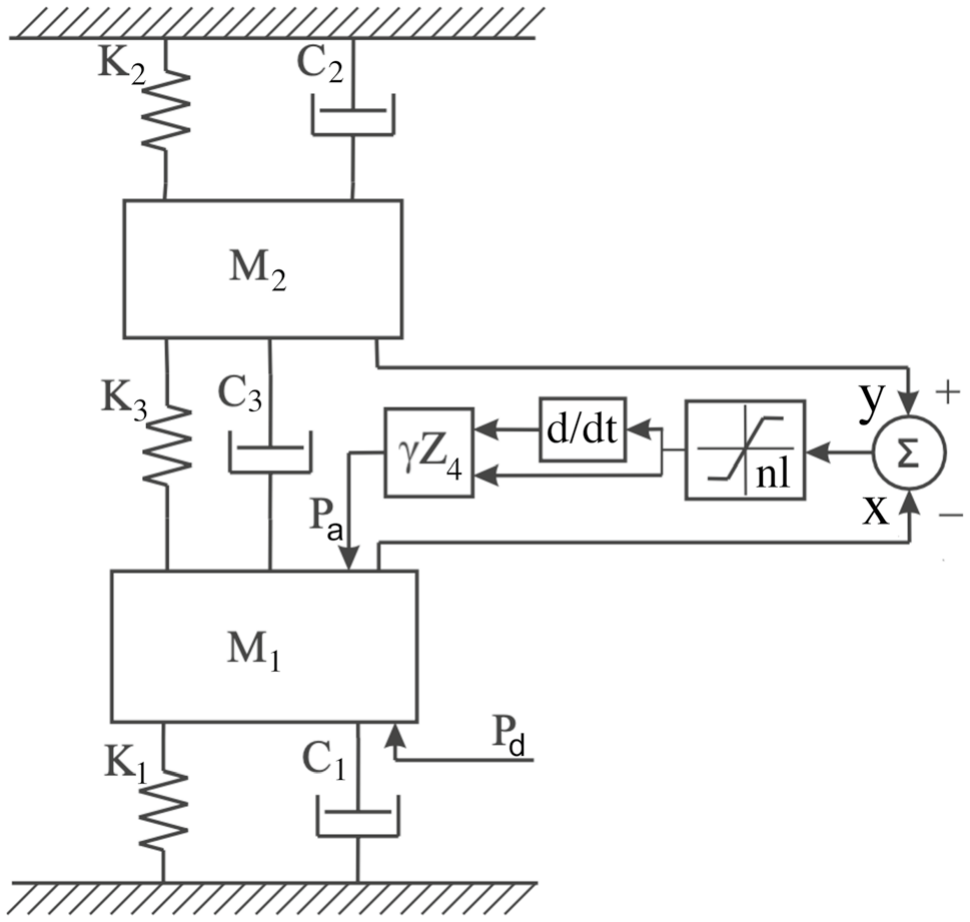
The Neely and Kim micro-mechanical element, as adapted by Elliott et al. $M_{1}$: mass of basilar membrane segment, $M_{2}$: mass of tectorial membrane segment, $K_{1,2,3}$: spring, $C_{1,2,3}$: damper, *x*, *y*: displacements of $M_1,M_2$, $P_d$: pressure difference, $P_a$: active pressure, *nl*: nonlinearity, $\gamma$: feedback gain, $Z_4$: impedance in feedback loop

$\ddot{x} +\omega (\alpha +\beta x^2-\gamma /\langle x^2 \rangle )\dot{x}+\omega ^2 x = 0$. The added term is an anti-damping term $\gamma /\langle x^2 \rangle$, in which $\langle x^2 \rangle$ is the square of the amplitude of *x* averaged over a “slow” time scale. To account for the very high mechanical sensitivity and sharp mechanical tuning in the results of in vivo measurements of cochlear-partition motion, Neely and Kim [49] modelled the cochlear partition as an array of two spring-mass-damper subsystems. To enhance tuning sharpness, each subsystem had a negative damping component. A few years later, the same authors replaced this subsystem with a three spring-mass-damper micro-mechanical element [50], to which Elliott et al. ([35], Fig. 11) added a saturating nonlinearity (labelled as “nl” in Fig. 7, which is a slightly adapted version of Fig. 3.4 in [38]).

Among other proposed multiple-element systems is the surface acoustic wave (SAW) model put forward by Bell [5]. This model proposes that positive feedback occurs between the rows of outer hair cells, which exhibit motility of both their stereocilia and hair cell bodies, leading to the formation of standing waves in the subtectorial space [51]. The resulting oscillation effectively supplies a sensor and a motor to each string of Gold’s “underwater piano” [4, 6], but the proposal requires further modelling and development.

### Intermezzo 2: Modelling Each of the Active Elements as a Compound Oscillator

Each of the 350 micro-mechanical elements in Fig. 3 is now represented by the elements set out in Fig. 7.

The equations to be solved for each element *j*, for the displacements $x_j(t)$ and $y_j(t)$ of masses $m_1$ and $m_2$ respectively, are adapted from Eqs. (30, 31, 33) in Elliott et al. [35]:

3 $$\begin{aligned} m_1 \ddot{x}_j(t)= a11_j\dot{x}_j(t)+a12_jx_j(t)+a13_j\dot{y}_j(t)+a14_jy_j(t)+p_{d,j}(t) \end{aligned}$$

4 $$\begin{aligned} m_2 \ddot{y}_j(t)= a21_j\dot{x}_j(t)+a22_jx_j(t)+a23_j\dot{y}_j(t)+a24_jy_j(t) \end{aligned}$$

with

$$a11_j=g\gamma _{0,j}c4(j)\gamma [x_j(t)-y_j(t),\dot{x}_j(t)-\dot{y}_j(t)]-(c1_j+c3_j)$$

$$a12_j=g\gamma _{0,j}k4(j)\gamma [x_j(t)-y_j(t),\dot{x}_j(t)-\dot{y}_j(t)]-(k1_j+k3_j)$$

$$a13_j=c3_j-\gamma _{0,j}c4(j)\gamma [x_j(t)-y_j(t),\dot{x}_j(t)-\dot{y}_j(t)]$$  

$$a14_j=k3_j-\gamma _{0,j}ck(j)\gamma [x_j(t)-y_j(t),\dot{x}_j(t)-\dot{y}_j(t)]$$  

wherein

$$\gamma [\alpha ,\beta ]=1-\tanh [(\alpha /\alpha _{sat})^2+(\beta /\beta _{sat})^2]$$

and with

$$a21_j=c3_j; a22=k3_j; a23=-(c2_j+c3_j); a24=-(k2_j+k3_j)$$

while $\varvec{p}_d(t)=a\varvec{F}^{-1}\varvec{\ddot{x}}(t)$, as in *Intermezzo 1*.

The values for the stiffness parameters *k* and the damping parameters *c*, for each of the 350 elements *j*, were taken from Table 4.1 in [38]. Other values: $m_1=0.044, m_2=0.0073$, lever gain $g=1$, damping $d_1=0.1$, angular frequency $\omega _1=2000\pi$, saturation values $\alpha _{sat}=2.10^{-5}$ and $\beta _{sat}=0.1$.

Equation $\ddot{x}_1(t)+d_1\omega _1\dot{x}_1(t)+\omega _1^2x_1(t)=p_{d,1}(t)$ for oscillator 1 was added to Eqs. (3) and (4), and the set of $n = 351$ equations was solved for a total time of 0.2s and a time step of 5$\mu$s.

Parameter $\gamma _0$ controls the damping of the vibration of all 350 masses *m*_1_; the larger $\gamma _0$, the smaller the damping. It was given the value 0.5 for all micro-mechanical elements, except for element 200: $\gamma _{0,200}=2.0$. This choice makes element 200 a self-sustaining oscillator, while all other elements are passive, comparable with the situation in *Intermezzo 1*.

**Fig. 8:**
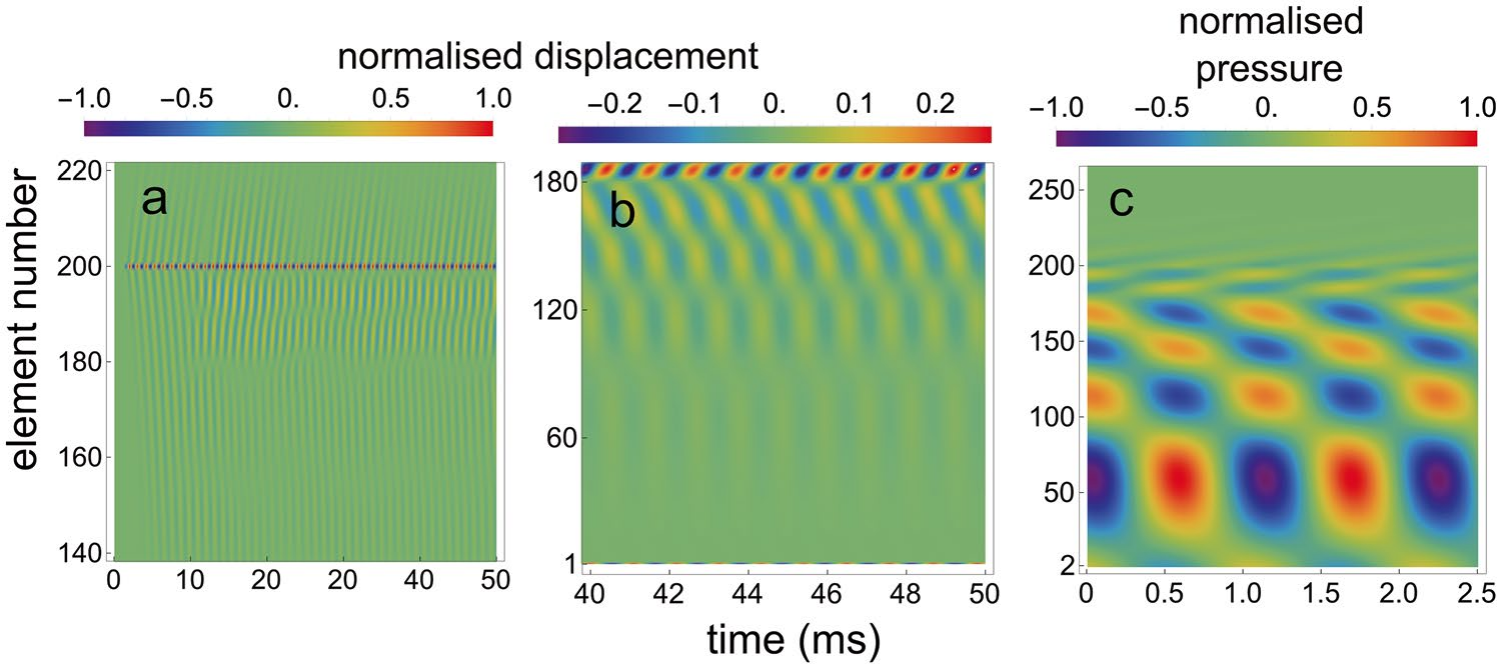
Density plots for one active element within a passive array. **a** For the normalized $x_j(t)$ for $j=$140 to 220, for the situation that oscillator 200 is self-sustaining. **b** For 40 to 50 ms after $t=0$, for $j=$1 to 185. The maximum amplitude in this panel is about 4 times smaller than the amplitude of oscillator 200. **c** For 2.5 ms of the normalized fluid pressure $p_{d,j}(t)$ for $j=$2 to 260, starting around $t=45$ ms after $t=0$

Initial values were $x_j(0)=0, y_j(0)=0,\dot{x}_j(0)=0, \dot{y}_j(0)=0$ for all *j*, except $\dot{y}_{200}(0)=0.0001$. Figure 8a and b are density plots for the calculated $x_j(t)$, for different time intervals and for different sets of oscillators. And Fig. 8c is a density plot for the calculated $p_{d,j}(t)$.

**Fig. 9:**
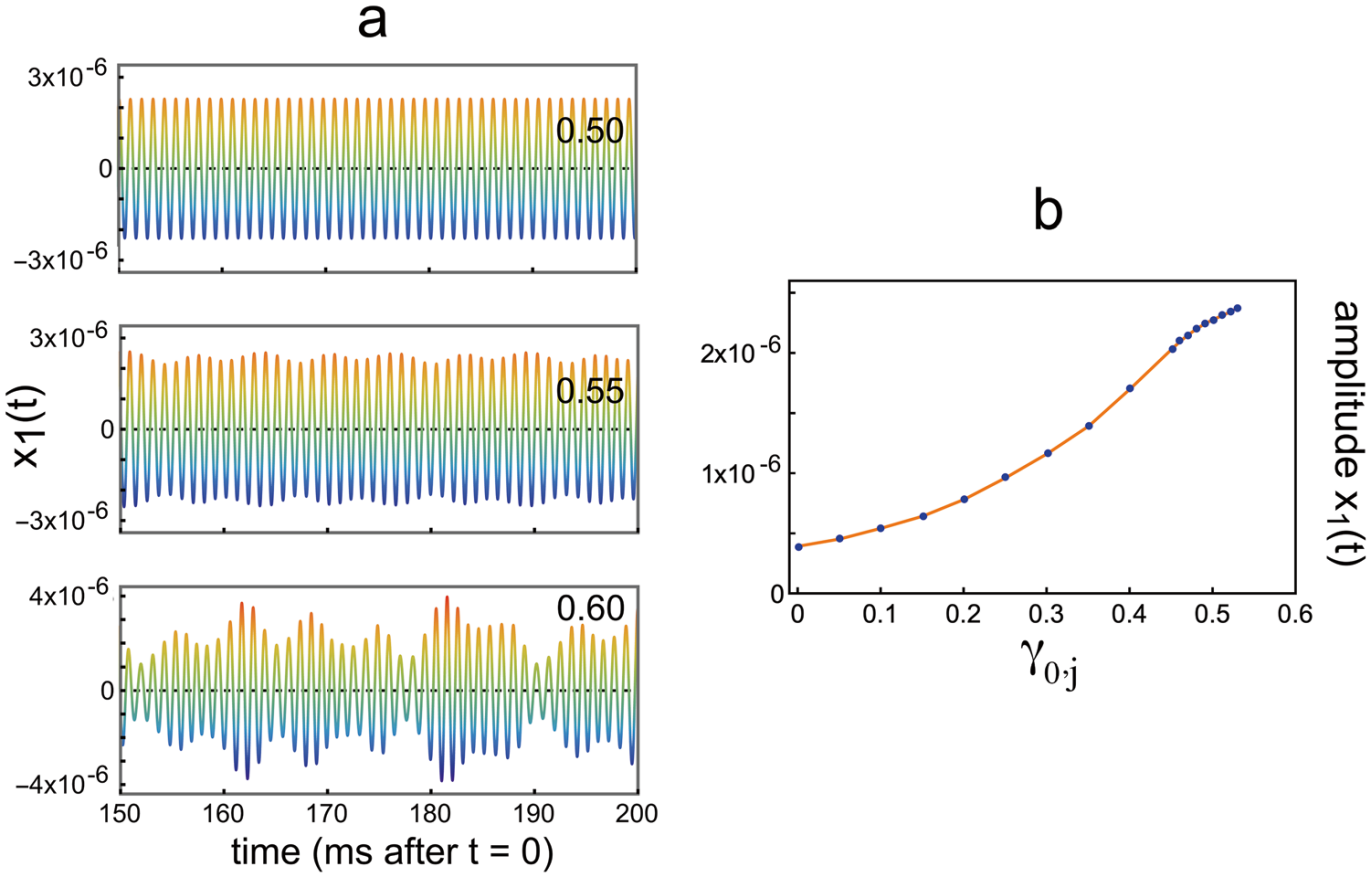
Effect of damping of the passive elements. **a** $x_1(t)$ for 3 values of $\gamma _{0,j}$, given in the upper right corner. **b** Blue dots: Amplitude of $x_1(t)$ for different values of $\gamma _{0,j}$. (The orange line connects the dots)

To show the effect of the value of $\gamma _0$ for the “passive” elements on the amplitude of oscillator 1, Fig. 9a gives $x_1(t)$ for 3 values of $\gamma _{0,j}$ ($j =2,..,199, 201,...,n$), while $\gamma _{0,200}$ remained at 2.0. Displacement $x_1(t)$ of oscillator 1 is no longer a sine wave with a constant amplitude for $\gamma _{0,j}>0.53$, ($j =2,..,199, 201,...,n$). And for $\gamma _{0,j}>0.6$ the system described by Eqs. (3) and (4) turned out to be unstable. This was not the case in *Intermezzo 1* above: $x_1(t)$ in Fig. 5 is a pure sine wave for all $\alpha _j$.

The amplitude of $x_1(t)$ for values of $\gamma _{0,j}$, for which $x_1(t)$ is a pure sine wave, was calculated for different values of $\gamma _{0,j}$, and is shown in Fig. 9b. When comparing this figure with Fig. 5c, it should be kept in mind that in the micro-mechanical element of Fig. 7 damping decreases with increasing feedback gain $\gamma$.

The conclusion from this numerical modelling is that the behaviour of this model — which also has one active oscillator — is not essentially different from that in Intermezzo 1. In both models, a standing fluid pressure wave is created between the active oscillator and oscillator 1. And in both models, the amplitude of the generated SOAE depends on the (un)damping of the passive oscillators or micro-mechanical elements. However, this effect is larger in Intermezzo 1 than in Intermezzo 2.

***End of Intermezzo 2***

## Introducing Time Delays and Irregularities

Strube [52] proposed cochlear Bragg [53] reflection at approximately periodic inhomogeneities to account for the long delays — roughly proportional to frequency — of click-evoked otoacoustic emissions (CEOAEs). He thought these inhomogeneities were possibly spatial variations of active undamping. Shera and Zweig [54] investigated Strube’s “washboarding” assumption, and concluded “ — because no orderly, periodic scattering structure has yet been found — that the microstructure of the primate organ of Corti is more chaotic than sinusoidal”. Later, Wit et al. [55] showed that random irregularities arranged all along the cochlear partition, are needed to produce realistic CEOAEs and their spectra [56]. More than 10 years earlier Sutton and Wilson [57] had already proposed a model in which emissions were caused by a few local irregularities in cochlear frequency mapping.

Speaking of the concept of irregularities, it is interesting to note that Gold, the predictor of (spontaneous) otoacoustic emissions [4], stated that if the tuning of individual elements in the inner ear overlapped perfectly, their outputs would ultimately cancel and that inaccuracies in the system are necessary to produce evoked sound [6].

Talmadge and Tubis [58] numerically investigated “instability modes”, created by the presence of inhomogeneities in a one-dimensional transmission line model for the mammalian cochlea. The model can — among other things — account for the periodicity in frequency spectra of evoked and spontaneous otoacoustic emissions (see Braun [59; Fig. 1A]).

Zweig and Shera [54, 60] explained this “striking periodicity” in the frequency spectra of evoked otoacoustic emissions as being the result of coherent reflection from random irregularities in the micromechanics of the organ of Corti. They proposed a novel scattering mechanism — an analogue of Bragg scattering in nonuniform disordered media — which creates (spectral) order out of spatial irregularity.

**Fig. 10:**
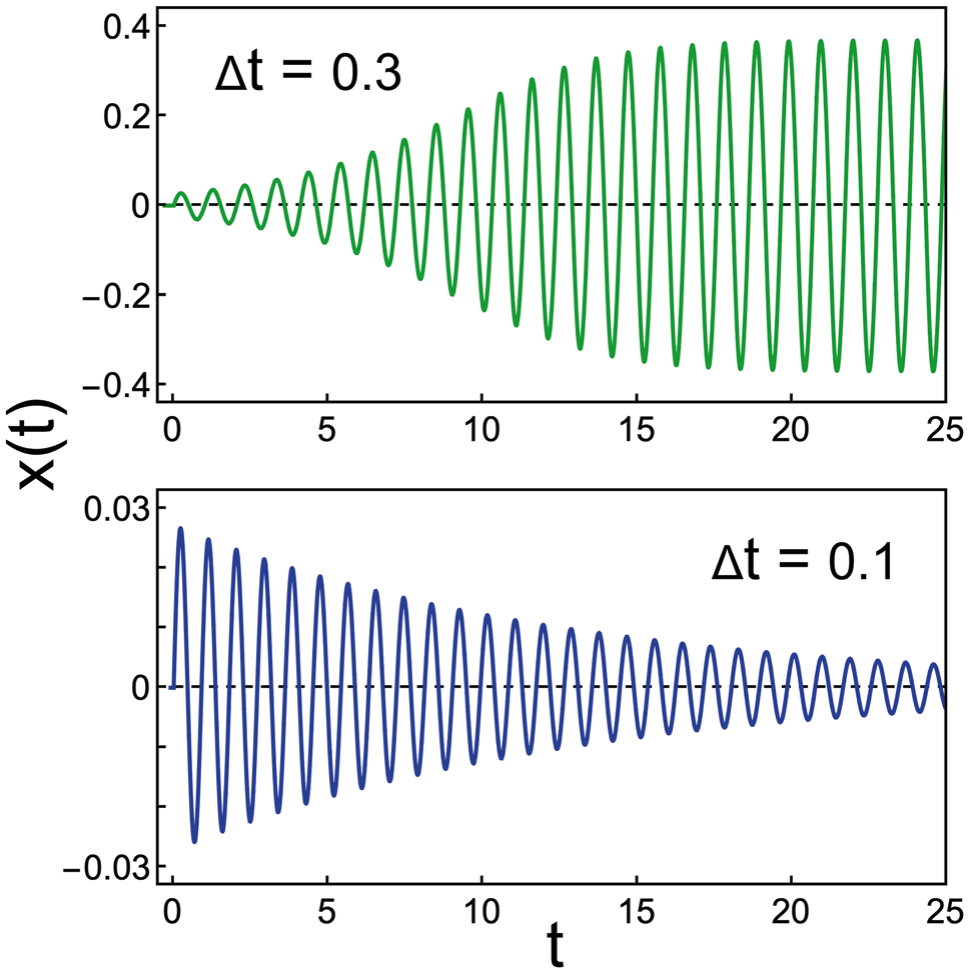
How introducing delayed feedback in a Van der Pol oscillator can change it from active (self-sustaining) to passive (damped). Displacement *x*(*t*) for the oscillator with equation $\ddot{x} +\omega (-\alpha +\beta x^2)\dot{x}+\omega ^2 (x(t) +\kappa x(t-\Delta t))= 0$, for two values of delay $\Delta t$; for $\omega = 2\pi$, $\alpha = -0.2$, $\beta = 3$, $\kappa = 0.3$, and $x(t, t \le 0)=0$, $\dot{x}(t, t \le 0)=0.2$

Talmadge et al. [61] elaborated Talmadge and Tubis’ [58] nonlinear time domain cochlear model with distributed basilar membrane roughness. The elaborated model used time-delayed stiffness, involving both slow and fast feedback of a form suggested by Zweig [62]; the slow feedback provided a source of negative damping and the fast feedback helped to create a tall and broad activity pattern, as seen in Mössbauer measurements of basilar membrane motion. The authors introduced delayed feedback in each of the basilar membrane elements by adding a $\Delta t$ term to the equation for the oscillator with a “Van der Pol -type” nonlinear damping function. Figure 10 illustrates how changing the delay can change the oscillator from active (self-sustaining) into passive (damped).

Shera and Guinan [63] argued that otoacoustic emissions arise from two fundamentally different mechanisms, the first being linear coherent reflection and the second being nonlinear distortion. In this dual-aspect process (illustrated in their Fig. 10), SOAEs are due to standing waves between random perturbations and the stapes, a process they called multiple internal coherent reflection. The details of this mechanism underlying the generation of mammalian SOAEs are set out in Shera’s global standing-wave model for the generation of mammalian SOAEs [64]. In this model, waves travelling forward from the stapes — initiated either by sounds from the environment or by internal noise — are partially reflected at impedance perturbations and travel back to the stapes, where they are reflected again. This process repeats itself several times, and all forward and backward travelling waves add up to produce standing waves. The first step of this process was already proposed by Kemp [15] in his reflection model to explain the generation of evoked emissions. In the active version of Shera’s model, standing wave amplitudes are actively maintained by wave amplification; in the passive version, they are driven by ongoing biological noise. Shera [64] compares the cochlear production of SOAEs in the cochlea with the coherent emission of light by an optical laser. This was also described earlier by Zweig [62], who writes that SOAEs are created by an oscillating biological “hydromechanical” laser.

The 1000 cochlear segments in the one-dimensional, active and nonlinear time-domain transmission line model of Epp et al. [65] can be described by a second-order differential equation with position- and velocity-dependent damping, position-dependent linear stiffness, and a velocity-dependent feedback stiffness term. The profiles of the velocity-dependent damping and stiffness terms are illustrated in their Fig. 1; the damping is negative for small velocities. To simulate the generation of SOAEs, a sustained and stable oscillatory activity was generated by introducing roughness into the model. Oscillation results from multiple reflected waves that are amplified by the negatively damped oscillators on the cochlear partition. Roughness is introduced in the stiffness term by multiplying it with $1+\epsilon .N(0,1)$ (with *N* being Gaussian noise with mean 0 and variance 1, and $\epsilon$ a scaling parameter).

The nonlinear time-domain model by Verhulst et al. [66] and Altoé et al. [67] accounts for both reflection and distortion source OAEs [61]. According to the authors, it simulates SOAEs through manipulation of middle ear reflectance, but no example is given of how the parameters of the model need to be chosen in order to make it generate this type of otoacoustic emission.

### Intermezzo 3: Modelling the Effect of One Irregularity

**Fig. 11:**
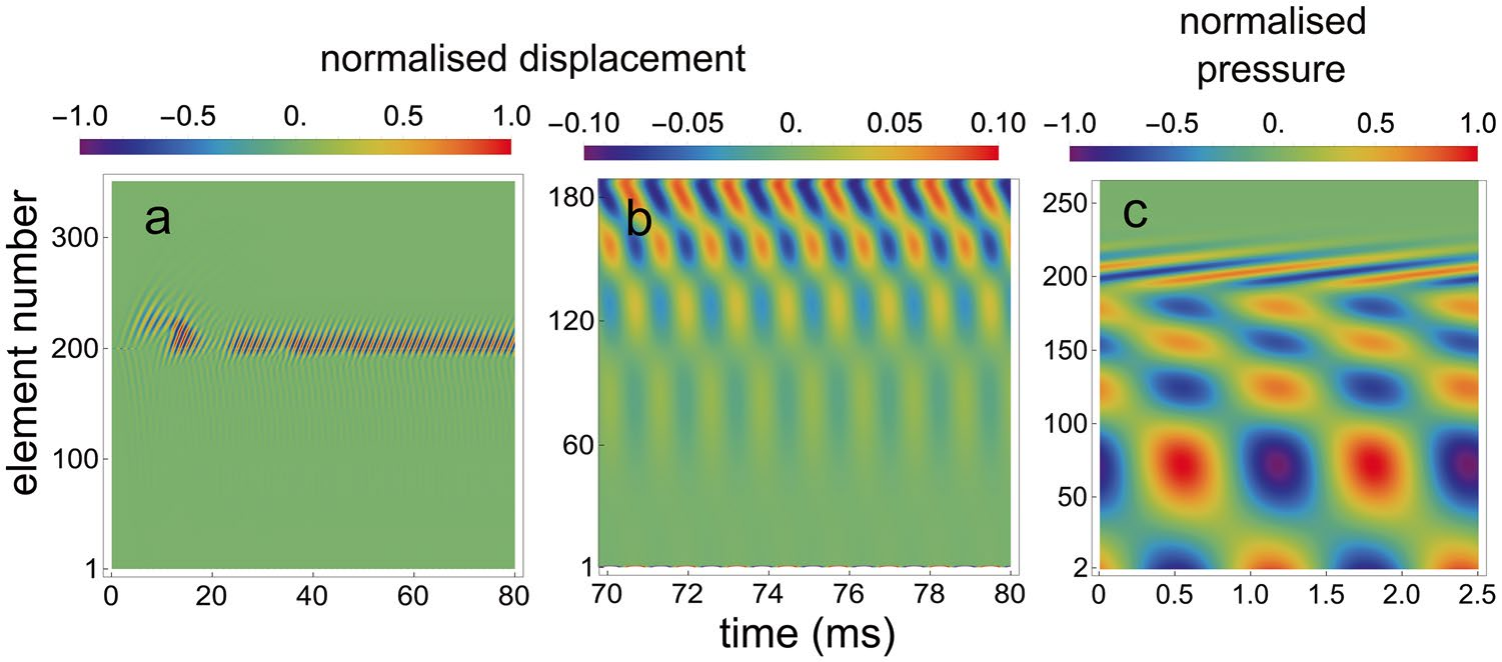
Density plots for a single irregularity embedded within an array of passive elements. **a** For the normalized $x_j(t)$ for $j=$1 to 350, where the damping of element 200 is somewhat larger than that of all other elements. **b** For 70 to 80 ms after $t=0$, for $j=$1 to 185. Maximum amplitude in this panel is 10 times smaller than the maximum amplitude in panel a. **c** For 2.5 ms of the normalised fluid pressure $p_{d,j}(t)$ for $j=$2 to 260, starting around $t=75$ ms after $t=0$

The calculations to yield Fig. 8 in *Intermezzo 2* are here repeated with the same values for the parameters and starting values, with the exception of $\dot{y}_{200}(0)=0.1$ and parameter $\gamma _0$. This feedback parameter was set at 0.65 for all elements, except for element 200. Instead of making this element the only active element, it was given a higher damping ($\gamma _{0,200}=0.5$), than the other elements. This created one irregularity in the array of micro-mechanical elements, resulting in the creation of a self-sustaining oscillation in the array, as can be seen in Fig. 11.

The striking result of the introduction of an irregularity into the array of elements is that it produces a self-sustaining oscillation and an SOAE at oscillator 1 (the middle ear and eardrum), even though the damping of all elements is so large that in isolation their impulse responses are damped oscillations. This result was earlier shown by Elliott et al. [35, Fig. 14], Ku et al. [36, Fig. 5] and Vignali et al. [68, Fig. 3]. These figures are comparable with Fig. 10a above.

**Fig. 12:**
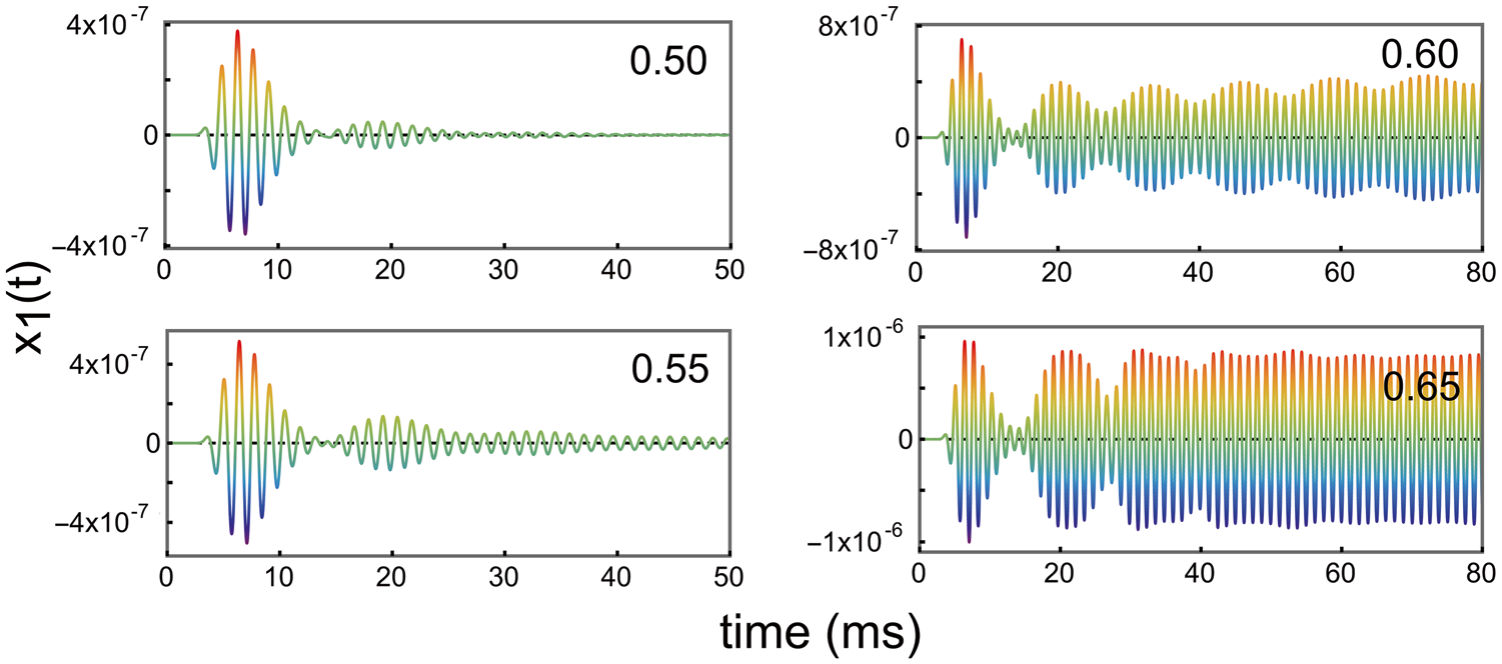
Displacement as a function of time for oscillator 1, for 4 values of $\gamma _{0,j}$ ($j =2,..,199, 201,...,n$), as given in the upper right corner. The parameter setting for the lower right corner ($\gamma _{0,j}=0.65$) is the same as that for Fig. 11

The modelling shows that if the feedback force that decreases the damping (controlled by parameter $\gamma _0$) is made too small, the presence of an irregularity at element 200 does not create a self-sustaining oscillation in the array of elements. This is illustrated in Fig. 12, wherein the calculated displacement as a function of time $x_1(t)$ for oscillator 1 is given for 4 values of $\gamma _{0,j}$ ($j =2,..,199, 201,...,n$), while $\gamma _{0,200}=\gamma _{0,j}-0.15,j\ne 200$.

It can be concluded from Fig. 12 that $\gamma _{0,j}$ ($j \ne 200$) has to be larger than 0.55 to create a self-sustaining oscillation in the array — that is, an SOAE at oscillator 1. It should be noted that this is precisely the condition for which $x_1(t)$ starts to deviate from a sine wave with constant amplitude in the situation with one active element, as we can conclude from inspection of Fig. 9 in *Intermezzo 2*.

This intermezzo illustrates that by introducing an irregularity, while keeping all elements of the model positively damped, a self-sustaining oscillation can be produced, as in Intermezzo’s 1 and 2. It also shows that the standing fluid pressure wave that creates the SOAE (Fig. 11c) is not essentially different from that in the two foregoing intermezzo’s. And last but not least, it is a prime example of how SOAEs are generated as a global collective phenomenon, “necessarily involving the mechanics, hydrodynamics, and cellular physiology of the entire cochlea, as well as the mechanical and acoustical loads presented to it by the middle and external ears” (as quoted from Shera [64]).

***End of Intermezzo 3***

## Introducing the Hopf Oscillator

**Fig. 13:**
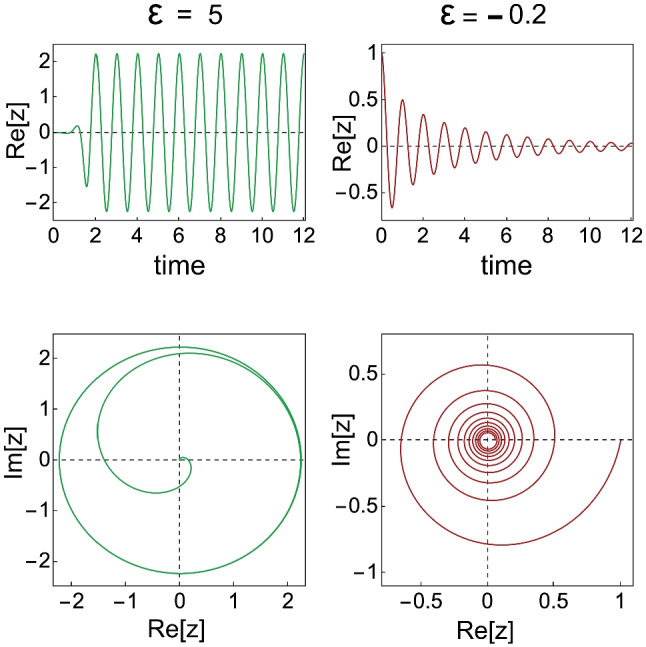
Characteristic features of a Hopf oscillator. Solutions of $\dot{z}=(i\omega _0+\epsilon )z-|z|^2z$ for $\omega _0= 2\pi$, for $\epsilon = 5$ (left column) and for $\epsilon = -0.2$ (right column). The lower panels show the corresponding limit cycles. A few cycles after $t=0$ the limit cycle for $\epsilon = 5$ is that for a pure sine wave; that for $\epsilon = -0.2$ spirals down to $z=0$

Following Gold’s [4, 6] arguments that the ear needs amplification to compensate for damping, Eguiluz et al. [69] showed how, by poising itself at a Hopf bifurcation, the cochlea could maximise tuning and amplification. The equation for a Hopf oscillator is: $\dot{z}=(i\omega _0+\epsilon )z-|z|^2z$, wherein *z*(*t*) is a complex variable of time, $\omega _0$ is the natural frequency of oscillation, and $\epsilon$ is a control parameter. Figure 13 gives solutions of the equation for two values of $\epsilon$. The transition from a self-sustaining to a damped oscillation is at $\epsilon =0$.

The solution *Re*[*z*] for $\dot{z}=(i\omega _0+\epsilon )z-|z|^2z$ is a pure sine wave for all $\epsilon >0$. This is in marked contrast with the characteristics of the Van der Pol oscillator, as can be seen by inspection of Fig. 1 (for $\alpha =1$).

Duke and Jülicher [70] combined the generalised version of a “self-tuned critical oscillator” with a travelling wave to establish a nonlinear active travelling wave in the cochlea. Their model generates basilar membrane displacement profiles in agreement with experimental observations.

In the same year (2003) Vilfan and Duke [71], motivated by how insect flight muscles act synchronously, investigated how an array of serially coupled Hopf oscillators can be synchronised by connecting them to an external load. For certain combinations of parameters, a self-sustaining oscillation can result, even though the isolated elements are themselves passive.

Inspired by this finding, it is shown in Intermezzo 4 how two oscillators, which are passive in isolation, can, when coupled, generate a self-sustained oscillation. This system can be taken as a fundamental version of the situation investigated in Intermezzo 3.

### Intermezzo 4: Two Coupled Hopf Oscillators

With the argument that a single oscillator model cannot adequately fit auditory nerve tuning curves, Lerud et al. [72] used pairs of coupled oscillators to model the dynamics of cochlear segments. In each of these pairs, one oscillator represented basilar membrane (BM) displacement dynamics, while the other represented organ of Corti (OC) dynamics.

If the two oscillators are bidirectionally coupled, and in the absence of an external stimulus, these dynamics are governed by [72, Eq. 9]:

$$\dot{z}_{bm}=z_{bm}(\alpha _{bm}+i2\pi f)+c_{12}z_{oc}$$,

$\dot{z}_{oc}=z_{oc}(\alpha _{oc}+i2\pi f+\beta |z_{oc}|^2)+c_{21}z_{bm}$, which both are the equations for a Hopf bifurcation.

The real parts of $z_{bm}$ and $z_{oc}$ are basilar membrane and organ of Corti displacements $x_{bm}(t)$ and $x_{oc}(t)$ respectively; $\alpha$ is a linear and $\beta$ is a nonlinear damping parameter; *f* is frequency; and $c_{12}$ and $c_{21}$ are coupling coefficients.

**Fig. 14:**
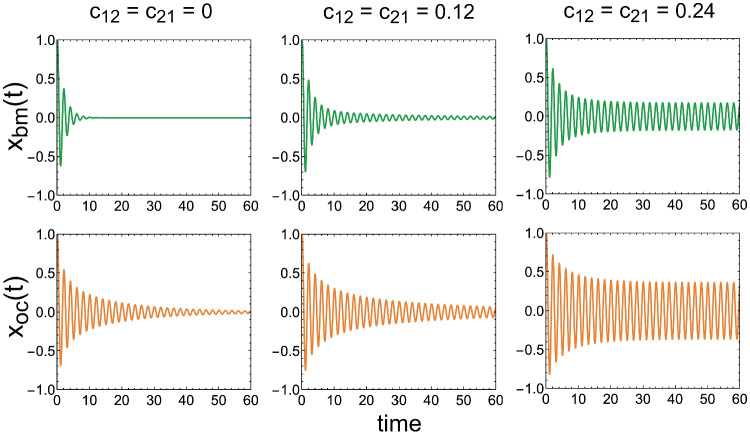
Impulse responses of a micromechanical element for three different values (columns) of mutual coupling strength parameters $c_{12}$ and $c_{21}$. The *y*-axes, $x_{bm}(t)$ and $x_{oc}(t)$, are the displacements of the individual basilar membrane and organ of Corti segments

The equations are solved for three values of $c_{12}=c_{21}$, for $f=0.5$, $\alpha _{bm}=-0.5$, $\alpha _{oc}=-0.05$, and $\beta =-0.5$ for initial values $z_{bm}(0)=z_{oc}(0)=1$. The results are shown in Fig. 14.

It is clear from this figure that in isolation (or when their coupling is weak) the BM segment, as well as the OC segment, behaves as a damped oscillator; but when coupled strongly enough the system behaves as a global oscillator.

***End of Intermezzo 4***

## A Brief Look at the Lizard Ear

Although it is not our main focus, it is worth looking briefly at work which endeavours to understand how SOAEs can be generated by the ears of lizards. Their ears have a totally different structure from mammalian ears, but there are some marked similarities in the behaviour of their SOAEs [3].

To model the generation of SOAEs in the ear of the bobtail lizard, Vilfan and Duke [73] added the term $+(d_R+id_I)(z_{j+1}-2z_j+z_{j+1})$ to the right side of $\dot{z}_j=(i\omega _j+\epsilon _j)z_j- B|z_j|^2z_j$, to couple the *j*-th oscillator in an array to its nearest neighbours. Depending on the value of the coupling terms $d_R$ and $d_I$, and of $\epsilon$ $(>0)$, the oscillators cluster into frequency plateaus [74]. In this interpretation, a single SOAE is represented by a cluster of frequency-locked oscillators.

Following Vilfan and Duke [73], Gelfand et al. [75] introduced nearest neighbour coupling in an array of oscillators to model SOAEs in the Tokay Gecko (a species of lizard). In their model, each oscillator represents a transverse row of hair cells, and in fact the oscillators are appropriately modelled as Van der Pol oscillators, characterised by the equations:

$\dot{x}_n=\mu _n(y_n-x_n^3/3+\alpha _nx_n)$ and $\dot{y}_n=[-(2\pi f_n)^2/\mu _n]x_n$, with $x_n(t)$ being the displacement of the *n*-th hair bundle. Combining the equations yields $\ddot{x}_n+\mu _n(-\alpha _n+x_n^2)\dot{x}+(2\pi f_n)^2x_n=0$, with parameters $\alpha$, *f*, and $\mu$ respectively determining amplitude, characteristic frequency, and nonlinearity of an individual unit’s unforced self-sustaining oscillation (for $\alpha >0$).

Gelfand et al. [75] investigated the viscous and elastic coupling of the oscillators separately. With viscous coupling, no clustering of the oscillators occurred, but with elastic coupling the 110 oscillators clustered in 15 frequency plateaus, for $\gamma = 1$ in the coupling term $\gamma (x_{n-1}-2x_n+x_{n+1})$.

The model by Vilfan and Duke [73] and that by Gelfand et al. [75] are strongly related. Each shows the entrainment of a cluster of oscillators by an external tone, provided that the tone is close to the intrinsic frequency of the cluster.

Wit and Van Dijk [76] employed the model of Vilfan and Duke [73] to investigate how several factors affected the behaviour of the oscillator array. The investigated conditions were frequency spacing, the value of coupling constants $d_R$ and $d_I$, values of effective damping $\epsilon$ and of intrinsic nonlinearity *B*, the presence of irregularities and noise, the presence of a discontinuity, and entrainment by an external tone. Of relevance to our survey, the authors concluded that this model can produce many well-established properties of human SOAEs.

## Two Detailed Models

**Fig. 15:**
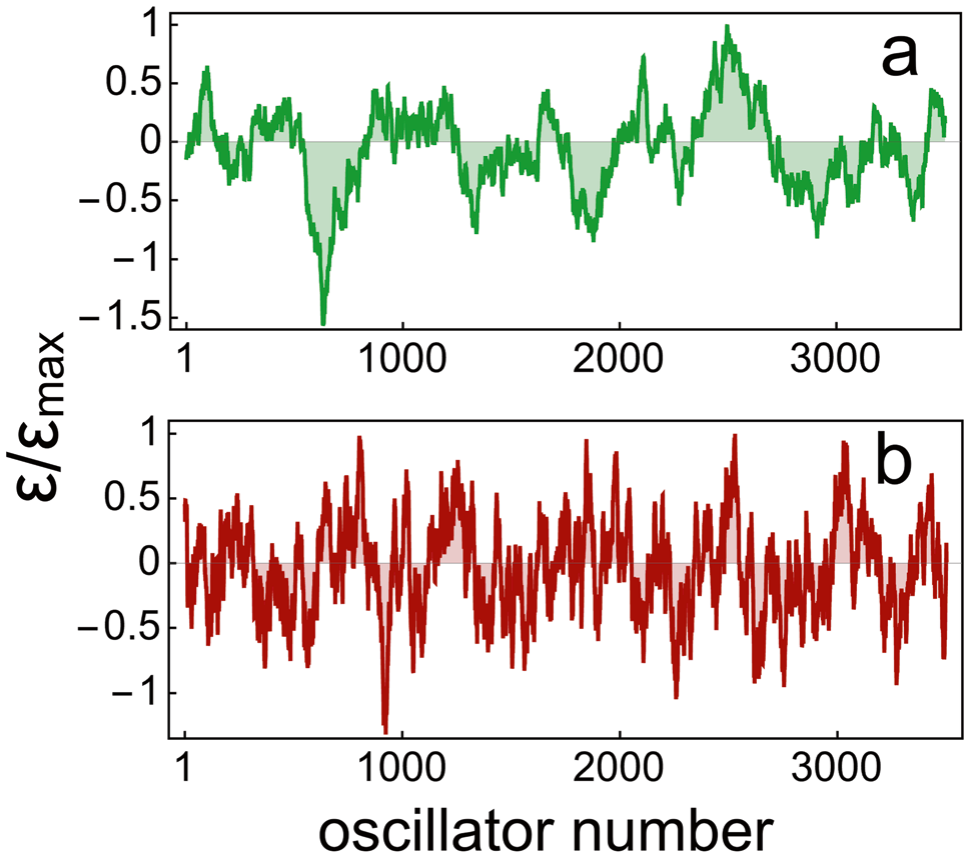
The distribution of cochlear imperfections parameter $\epsilon (x)$, generated with Mathematica command “OrnsteinUhlenbeckProcess”, for mean reversion speed 0.008 (panel **a**) and 0.04 (panel **b**)

Fruth et al.’s [77] active oscillator model to describe the statistics of human SOAEs is the extension of the model proposed by Duke and Jülicher [70]. Each of the 3500 oscillators in Fruth’s model obeys the generalised complex Ginzburg-Landau equation:

$\dot{z}=[\epsilon (x)+i\omega (x)]z-\beta |z|^2z+(\kappa +i\kappa '){{d^2z}\over {dx^2}}-{i\over \alpha }p+\xi (x,t)$, with $\epsilon , \omega , \beta , \kappa , \kappa '$, and $\xi$ having the same meaning as the corresponding parameters in Eq. (11) in Vilfan and Duke’s model [72]; while *p* is the fluid pressure difference across the basilar membrane (BM), and $\alpha \omega (x)$ is the local static BM stiffness. Cochlear imperfections are described by static spatial variations of bifurcation parameter $\epsilon$, generated by an Ornstein–Uhlenbeck process, as illustrated in Fig. 15.

Panel a in Fig. 14 resembles Fig. 1B in the model by Fruth et al. [77]. SOAE spectra, calculated with this model, are in frequency regions where the oscillators are active ($\epsilon (x)>0$). According to the authors, emissions are not caused by individual oscillators, but from the synchronisation of groups of active oscillators, a phenomenon that depends on *global* features of the system. The irregularity in $\epsilon (x)$ is supposed to be stable over time and unique to each ear, giving each cochlea its individuality.

The physics-based model for the generation of mammalian SOAEs by Meaud et al. [78] used a finite element model of the cochlea developed earlier [79–81]. As with Shera’s [64] coherent reflection model, it calls on cochlear roughness to produce SOAEs. The model included a three-dimensional treatment of the acoustics of the intra-cochlear fluid ducts, a micro-mechanical model of the vibration of the cochlear structures, and a nonlinear model of OHC biophysics. Roughness is introduced by multiplying model parameter $\epsilon _3(x)$ with $1+\Delta R\times r(x)$, where $\Delta R$ is a dimensionless number, *r* a random number taken from a standard normal distribution, and *x* the position along the basilar membrane’s longitudinal axis. Parameter $\epsilon _3$ is the electromechanical coupling coefficient in Eq. (6) in [80] for outer hair cell force and current. Changing parameter $\epsilon _3$ is similar to changing feedback parameter $\gamma _0$ in *Intermezzo 3* above; this parameter controls the feedback pressure that determines the damping of a micro-mechanical element, as can be concluded from inspection of Fig. 7.

## General Discussion: Local or Global?

SOAEs are the result of self-sustaining oscillations of the tympanic membrane, which in turn are generated by self-sustaining periodic changes in the pressure of cochlear fluids acting on the oval window. Accordingly, all the models for generating SOAEs reviewed in the preceding sections are made up of a macroscopic part (a fluid-filled box), and an array of micro-mechanical elements. Each element in the array is an oscillator with its own feedback force, a force that partly or completely compensates for the damping force(s) acting on the oscillating mass(es). This compensating device is called the *cochlear amplifier* (see for instance Section 5.4 in Duifhuis’ book on cochlear mechanics [82]). Displacement of the individual elements is coupled to the round window by the fluid, while — at the same time — fluid pressure acts on the elements.

Shera [64, 83] compares two alternative models for the origin of mammalian SOAEs: the global standing-wave model and the local-oscillator alternative. In his 2003 key publication, this author distinguishes between these two models in the following way [64, page 245]:

“In the local-oscillator model, the macro-mechanical structures and processes play no fundamental role, they serve merely to connect the autonomous oscillating element with the external environment, providing a conduit for the acoustic energy it produces to escape from the inner ear. In the global standing-wave model, by contrast, the oscillating element comprises the entire cochlea, and the collective response of the hearing organ as a whole contributes essentially to creating, maintaining, and determining the characteristics of the emission”.

Following Shera’s description of a local oscillator model, it is clear that the only active oscillator in *Intermezzo 1* is “the autonomous oscillating element.” However, the other passive oscillators are not simply “conduits for the acoustic energy to escape from the inner ear”. Changing their (un)damping — the cochlear amplifier — will change the amplitude of the SOAE at oscillator 1 by as much as a factor of 16 (see Fig. 5c).

In *Intermezzo 2* the micro-mechanical elements, being Van der Pol oscillators in *Intermezzo 1*, were replaced by the elements (two coupled masses) of Fig. 7. Here, again only one element is active, and this is the only local oscillator. Comparing Figs. 4 and 7, we learn that the change of elements did not essentially change the behaviour of the model — with one exception. That is, above a certain value of the (un)damping parameter for the passive elements the system became unstable (see Fig. 9). This restricts the range of amplification to a factor of 6.

The entire system of Fig. 3 behaves as a global oscillator in *Intermezzo 3, * generating an SOAE at oscillator 1, being a “reflection source emission” in Shera’s terms [64]. And yet, the system was changed from a local oscillator model in *Intermezzo 2* into a global oscillator (in *Intermezzo 3*), only by changing the (un)damping parameter $\gamma _{0,j}$ of the micro-mechanical elements.

Again citing Shera [64], page 245], reflection source emissions are generated as follows: “Backward-traveling cochlear waves are generated by the coherent scattering of forward-traveling waves off densely and randomly distributed perturbations in the mechanics of the cochlea”. Furthermore: “The resulting backward-traveling waves are then reflected by the impedance mismatch at the cochlear boundary with the middle ear, generating additional forward-traveling waves that subsequently undergo another round of coherent reflection near their characteristic places. At frequencies for which the total phase change due to round-trip wave travel is an integral number of cycles, standing waves can build up within the cochlea, which is then acting, in effect, as a tuned resonant cavity. Cochlear standing waves can become self-sustaining — and thus appear in the ear canal as spontaneous emissions — when the total round-trip power gain matches the energy losses, e.g., from viscous damping and acoustic radiation into the ear canal experienced en route”.

This description of the generation of a so-called reflection source emission requires the generation of a self-sustaining standing wave by *coherent* reflection from *multiple* perturbations. But as we saw in *Intermezzo 3* (Fig. 10b and c), a standing wave can also be generated by reflection from just one discontinuity. Furthermore, a standing wave with the same characteristics as that in Fig. 10 has been generated by a single local oscillator model (Figs. 4 and 8). Also in Intermezzo’s 1 and 2, it was shown that the model volume between the discontinuity (in this case the single active oscillator) and the stapes (oscillator 1) behaves as a resonant cavity, in which the amplitude of the standing wave depends on the (un)damping (“amplification”) of the micro-mechanical elements in this volume.

This suggests that the difference between a global and a local oscillator model might only reflect a difference between the type of discontinuity. In the global model there are discontinuities in the array of natural frequencies and/or some other parameter. In the local oscillator model, the active oscillators are the result of discontinuities in the array of damping parameters, which eventually combine with other discontinuities. But another difference between the two models should not be overlooked: the creation of a standing wave is essential in the global model. On the other hand, a local oscillator model would also generate an SOAE if no standing wave is created, because of a perfect match in the mechanical coupling between the cochlea and middle ear for the frequency of the SOAE. This was long ago illustrated by Van Hengel et al. [33]. (Compare their Figs. 6 and 8a).

An example of a model where the distinction between global and local is difficult to make is the model of Fruth et al. [77], as briefly described in the “Two Detailed Models” section. In this model, SOAEs are only present in basilar membrane regions where the individual oscillators are active. This serves as a reminder of the fact that no synchronised spontaneous OAEs (SSOAEs) are present in hearing loss ranges in impaired ears, as found by Sisto et al. [84]. This suggests — according to the authors — that the correlation between the presence of long-lasting OAEs and good cochlear functionality is local in the frequency domain. In other words, it can be interpreted as being local on the basilar membrane place-frequency map.

## Conclusion

After almost half a century from the discovery of SOAEs, and more than 70 years since they were first conjectured, the debate about how to best model them is still going on, as can be concluded from abstracts submitted for the 46th Midwinter Meeting of The Association for Research in Otolaryngology [85], and for the 184th meeting of the Acoustical Society of America [86]. Both Meaud et al. [85] and Samaras et al. [86] compare a (local) model based on coupled limit-cycle oscillators with a (global) model based on self-sustaining standing waves.

We have focussed on models for the generation of SOAEs in the mammalian cochlea. All these models have in common that micro-mechanical elements create self-sustaining oscillations as the result of mechanisms that reduce — or completely compensate for — damping. They reflect what Gold imagined back in 1948 — that the human ear functions like a piano immersed in fluid, with each string made active by equipping it with a regenerative receiver. Such a basic model of course benefits from multiple refinements, as surveyed here, but Gold would probably agree we are on the right track.

## Data Availability

On request to corresponding author.
