# Correction: Something in Our Ears Is Oscillating, but What? A Modeller’s View of Efforts to Model Spontaneous Emissions

**DOI:** 10.1007/s10162-024-00951-4

**Published:** 2024-05-20

**Authors:** Hero P. Wit, Andrew Bell

**Affiliations:** 1 Department of Otorhinolaryngology/Head and Neck Surgery, University of Groningen, University Medical Center Groningen, Groningen, Netherlands; 2 Graduate School of Medical Sciences, Research School of Behavioural and Cognitive Neurosciences, University of Groningen, Groningen, Netherlands; 3 John Curtin School of Medical Research, The Australian National University, Canberra, Australia

**Correction to: Journal of the Association for Research in Otolaryngology** 10.1007/s10162-024-00940-7

The original online version of this article was revised to correct the presentation of two equations in the section Intermezzo 2: Modelling Each of the Active Elements as a Compound Oscillator. The revised equations should read:

$${a13}_{j}={c3}_{j}-{\gamma }_{0,j}c4(j)\gamma[x_{j}(t)-y_{j}(t),\dot{x}_{j}(t)-\dot{y}_{j}(t)]$$

$${a14}_{j}={k3}_{j}-{\gamma }_{0,j}ck(j)\gamma[x_{j}(t)-y_{j}(t),\dot{x}_{j}(t)-\dot{y}_{j}(t)]$$

A sentence in the introductory section Early Days was also revised to read:

Step by step, we discuss novel contributions, punctuated now and then by numerical simulations whose goal is to illustrate key points.

The original article has been corrected.

